# The functional analysis of NirBD in microorganisms and the efficacies of NirBD on the soil nitrogen storage and N_2_O emission

**DOI:** 10.1016/j.synbio.2025.12.017

**Published:** 2026-01-15

**Authors:** Yidan Peng, Tengxia He, Qimin Zhou, Mengyuan Yin, Chengtao Jin

**Affiliations:** a Key Laboratory of Plant Resource Conservation and Germplasm Innovation in Mountainous Region (Ministry of Education), Guizhou Key Laboratory of Agricultural Microbiology, College of Life Sciences, Guizhou University, Guiyang, Guizhou Province, 550025, China; b Key Laboratory of Karst Georesources and Environment (Guizhou University), Ministry of Education, Guizhou University, Guiyang, Guizhou Province, China

**Keywords:** NirBD, Soil nitrogen storage, N_2_O emission, Microorganisms, DNRA and denitrification

## Abstract

The siroheme-containing nitrite reductase (NirBD), which is encoded by the *nirBD* gene, is a functional enzyme in the nitrogen cycle. The NirBD enzyme can regulate N_2_O release through the two distinct pathways of assimilatory nitrate/nitrite reduction to biomass and dissimilatory nitrate/nitrite reduction to ammonium. Therefore, a thorough comprehension of the function of NirBD in microorganisms can enable us to better understanding the contributions for nitrogen retention and N_2_O emission. However, the knowledge of the functions and expression mechanisms of *nirBD* gene across different microorganisms remains limited. This review synthesized the current research on the phylogenetic distribution and catalytic versatility of NirBD in fungi, bacteria, and actinomycetes. The contributions of NirBD for nitrogen retention and N_2_O emission were extensively discussed under anaerobic and aerobic conditions. The expression mechanism of NirBD was demonstrated. The factors that affect the expression amount of the NirBD enzyme in microorganisms were clarified systematically. This review not only elucidated the unique role of NirBD in the nitrogen metabolism but also provided a critical theoretical foundation for developing future strategies to enhance soil nitrogen fertility and mitigate N_2_O emissions.

## Graphical abstract

**Figure:**
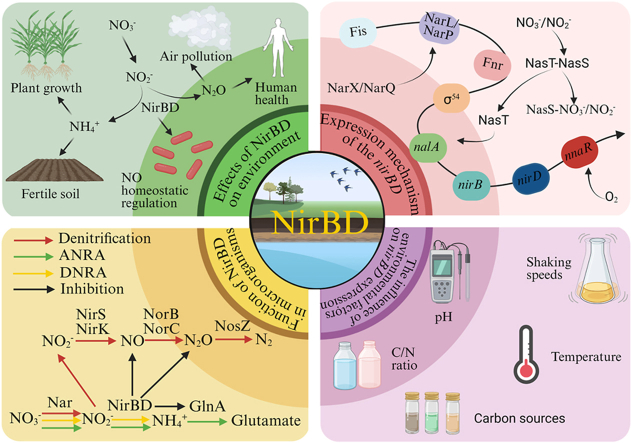

## 1 Introduction

Nitrogen serves as a fundamental biological element and a primary limiting nutrient for terrestrial life, forming an essential requirement for all organisms [1]. However, excessive nitrogen fertilizer application and poor crop assimilation efficiency leads to substantial nitrogen losses from agricultural soils in the form of greenhouse gases. Concurrently, the unchecked discharge of nitrogen-rich industrial wastewater exacerbates eutrophication, which degrades water quality, causes aquatic mortality, and poses significant threats to both human health and ecosystem integrity [2]. Microbial communities play crucial roles in both nitrogen retention within terrestrial systems and nitrogen removal from aquatic environments. The biological nitrogen cycle encompasses six distinct transformation pathways by prokaryotes, including nitrogen fixation (N_2_ → NH_4_^+^), ammonification (organic N → NH_4_^+^), nitrification (NH_4_^+^ → NO_2_^−^ → NO_3_^−^), denitrification (NO_3_^−^ → NO_2_^−^ → NO → N_2_O → N_2_), anaerobic ammonium oxidation (anammox: NO_2_^−^ → NH_4_^+^ →N_2_), and dissimilatory nitrate reduction to ammonium (DNRA: NO_3_^−^ → NO_2_^−^ → NH_4_^+^) [3]. Fungal species contribute primarily to oxygen-dependent nitrogen transformations, including nitrification, denitrification, and DNRA [4]. The partitioning of these microbial nitrogen transformations is governed by oxygen availability, with nitrification requiring aerobic conditions, while denitrification and DNRA prevail in anaerobic or microaerophilic environments.

The NirBD enzyme is a pivotal catalyst in the nitrogen cycle, involved in assimilatory nitrate reduction to ammonium (ANRA), dissimilatory nitrate reduction to ammonium (DNRA), and the modulation of N_2_O emissions [5]. The soluble NirBD heterodimer assembles from the catalytic NirB and structural NirD subunits, which are encoded by the *nirB* and *nirD* genes, respectively. This complex contains dual nucleotide-binding domains, an iron-sulfur cluster, and a siroheme prosthetic group [6]. In fermentative bacteria, NirBD participates in anaerobic redox balance by regenerating NAD^+^ to sustain glycolysis, concomitantly generating supplementary ATP through substrate-level phosphorylation during acetate metabolism [7]. Through such functional mechanisms, the enzymatic complex of NirBD enhance the soil nitrogen conservation and mitigates greenhouse gas emissions. The process of ANRA can facilitates the conversion of nitrate/nitrite to ammonium, which is subsequently channeled into glutamate biosynthesis for cellular nitrogen assimilation and growth. DNRA process is a microbial respiratory process that reduces nitrite to ammonium, thereby conserving nitrogen as a readily available nutrient for the microbiota and plants. Therefore, both DNRA and ANRA processes can enhance terrestrial nitrogen retention by diverting nitrogen from the denitrification pathway, thereby suppressing atmospheric N_2_O release.

While denitrification is the primary source of N_2_O, a subset of DNRA microorganisms has been identified as a non-canonical source of this greenhouse gas. For instance, the strain *Bacillus paralicheniformis* LMG 6934 could convert up to 15 mmol/L nitrate primarily to ammonium (>85 %), accompanied by minor N_2_O emission (<15 %) and no nitrite accumulation [8]. Similarly, N_2_O accounted for only approximatedly 0.15 % of the nitrate consumed by *Wolinella succinogenes* during exponential growth [9]. The presence of the *nirBD* gene was confirmed in both *Bacillus paralicheniformis* LMG 6934 and *Wolinella succinogenes*. In contrast, the canonical denitrifying bacterium *Pseudomonas aeruginosa* exhibited notable N_2_O production on carbon felt electrodes, with a conversion efficiency from nitrate to N_2_O exceeding 80 % [10]. Compared with the high N_2_O producing denitrifier of *Pseudomonas aeruginosa*, both *Bacillus paralicheniformis* LMG 6934 and *Wolinella succinogenes* emitted substantially less N_2_O*.* However, the role of the *nirBD* gene cluster in N_2_O production was demonstrated by its knockout in *Pseudomonas putida* Y-9, which drastically reduced N_2_O emissions under nitrate/nitrite respiration [11]. This role was further supported by the finding that its up-regulation promoted N_2_O accumulation [12]. In summary, the regulatory role of *nirBD* is intricately regulated by various factors, yet the precise molecular mechanisms governing its contribution to N_2_O fluxes remain elusive.

This review was designed to provide a comparative analysis of the functional roles of *nirBD* across bacterial, fungal, and actinomycete systems. Building on this foundation, it sought to integrate these roles within ANRA, DNRA, and nitrogen gas flux pathways to establish a mechanistic link governing soil nitrogen retention and N_2_O emissions. Furthermore, the expression mechanisms of *nirBD* and its function in the core nitrogen metabolic networks were clarified. Collectively, these insights would provide a theoretical basis for leveraging microbial nitrogen storage strategies, with potential applications in enhancing soil nitrogen sequestration and mitigating N_2_O emissions from denitrification processes.

## 2 Functional analysis of NirBD in microorganisms

### 2.1 Functional analysis of NirBD in bacteria

The DNRA of bacteria is a two-stage biogeochemical pathway, catalyzed stepwise by distinct enzymes. In contrast to denitrification processes, The DNRA pathway directly reduces nitrate to ammonium via nitrite without intermediate gaseous nitrogen species. Bacterial DNRA is mediated through two principal routes: the fermentative DNRA (F-DNRA) pathway and the respiratory DNRA (R-DNRA) pathway. The predominance of each pathway is governed by the type of microorganisms, substrate availability, and cellular energy conservation strategies [13]. The reduction of nitrite to ammonium is catalyzed by the enzymes encodes by four key functional genes: *nirB*, *nirD*, *nrfA*, and *nrfH*. Biochemical analyses revealed that the NADH-dependent NirBD complex serves as the catalytic subunit for F-DNRA, whereas the cytochrome *c*552-linked NrfAH system facilitates R-DNRA [14]. Recent thermal response studies demonstrated that the abundance of the DNRA genes of *nirB* and *nirD* increased during heating and high temperature stages in composting whereas a gradual decrease was observed during the cooling stages. In contrast, the abundance of *nrfA* gene showed the opposite trend [15]. From a bioenergetic perspective, these pathways employ distinct ATP generation mechanisms. The respiratory DNRA harnesses the proton motive force from oxidative phosphorylation to drive nitrite reduction, whereas fermentative DNRA is characterized by direct ATP generation via substrate-level phosphorylation [16]. The DNRA process features an initial respiratory step for nitrate reduction, while the overall pathway diverges into respiratory or fermentative types, governed by the specific bacterium and its growth substrates [17].

**Fig. 1:**
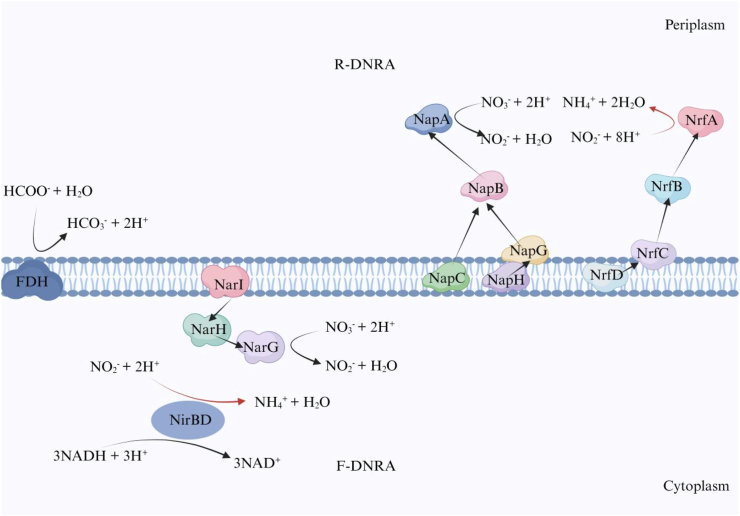
Comparison of the mechanism of nitrate conversion to ammonium in F-DNRA in bacteria under high nitrate concentration and R-DNRA in *Escherichia coli* under low nitrate concentration.

The spatial organization and catalytic mechanisms of the DNRA enzyme systems were shown in Fig. 1, which highlighted two distinct nitrate-nitrite reductase configurations: the periplasmic Nap/Nrf system and the cytoplasmic Nar/Nir complex [18]. Membrane topology analysis revealed that the Nar/Nir system consists of:

(1) *The narGHJI* operon-encoded nitrate reductase subunits (NarG, NarH, NarJ, NarI), which form a membrane-anchored oxidoreductase complex [19].
(2) *The nirBDCysC*-encoded soluble cytoplasmic nitrite reductases (NirB, NirD, NirC, NirycC), which mediate substrate-level phosphorylation [20].

When the concentration of nitrate in the substrates where bacteria are located is high, the membrane-bound nitrate reductase subunit complex (NarGHI) couples the reduction of nitrate with the oxidation of NADH by NADH dehydrogenase, forming an electron transport chain (ETC) on the cytoplasmic side of the bacterial plasma membrane [21]. This ETC generates a proton motive force across the membrane, thereby driving the ATP synthesis via chemiosmosis [22]. NarG in the ETC acts as a catalytic subunit and reduces nitrate to nitrite [23]. The generation of NO_2_^−^ is transported into the cytoplasm, and subsequently reduced to ammonium by the soluble NADH-dependent nitrite reductase NirBD through substrate-level phosphorylation [24]. Nir and Nar collectively accounted for 80 % of the total nitrite reduction, with the residual 20 % being mediated by NrfA [25].

Respiratory DNRA process exhibits two hallmark characteristics: independence from fermentable organic substrates (e.g., formate) or inorganic salt electron donors, and the energy conservation through electron transport chain (ETC)-coupled oxidative phosphorylation [26]. *Wolinella succinogenes, a rumen bacterium,* serves as the best-characterized model organism for this process owing to its capacity for facultative anaerobic growth driven by nitrate respiration with H_2_ or formate as the electron donors. The strain of *Wolinella succinogenes* contained only a Nap/Nrf combination of nitrate reductase and nitrite reductase, the expression of which was specifically induced under low nitrate conditions [21]. As detailed in Fig. 1, the nitrate reduction module comprises the NapAGHBFLD complex, in which NapA serves as the catalytic subunit for catalyzing the reduction of nitrate to nitrite. Through the electron transfer chain, periplasmic nitrite is reduced to ammonium via NrfA-mediated oxidative phosphorylation. This mechanism allows nitrite to serve as a terminal electron acceptor, thereby promoting anaerobic respiration while retaining nitrogen in a bioavailable form [13]. In addition to the peripheral NrfA, several crystal structures of the NrfA protein have also been discovered, which may be encoded by certain *nrfA* genes [27]. These crystal structures are proposed to catalyze the sequential reduction of nitrite to nitric oxide (NO), followed by the further reduction of NO to ammonium and nitrous oxide (N_2_O) via ancillary enzymes. Mechanistically distinct from the NrfA pathway, the nitrite reductase (NirS and NirK), which produce NO via denitrification pathway do not enter the periplasmic NrfA pathway due to the positive redox potential of its cofactors. Therefore, NO is considered the critical point at which DNRA and denitrification diverge. Beyond its role in nitrogen respiration, NrfA also demonstrates the capacity to reduce sulfite to sulfide (S^2−^), suggesting a potential functional link between this periplasmic enzyme and the biogeochemical cycling of sulfur [28].

NirBD is the main enzyme that converts nitrite to ammonium in the DNRA and ANRA pathways [29]. The ANRA pathway initially converges with DNRA in the stepwise reduction of nitrate to nitrite and then to ammonium, but critically diverges by channeling the ammonium directly into cellular biosynthesis via the glutamine synthetase/glutamate synthase (GS/GOGAT) system [30]. NirBD can modulate the ammonium assimilation by down-regulating the expression of *glnA*, which encodes glutamine synthetase [11]. When energy is limited and nitrate serves as the sole nitrogen source, ANRA represents a more favorable metabolic strategy than DNRA for optimizing nitrogen assimilation. This metabolic preference is driven by the substantial energy demand of DNRA, which requires two additional moles of electrons per nitrate molecule reduction compared to the more energy-efficient ANRA pathway [31]. In the nitrate respiration process, NarG subunits frequently undergo Hcp-dependent S-nitrosylation, a redox-sensitive modification that induces conformational changes and mediates protein-protein interactions among nitrate reductase, formate dehydrogenase, and the NirBD complex [32]. Genetic evidence from mutant strain analyses revealed that simultaneous disruption of nitrite genes (Δ*nirB* or Δ*nrfA*) and NO detoxification systems (Δ*norVW*/Δ*hmp*) caused severe growth impairment in minimal media, due to a failure in maintaining NO homeostasis. This model was further supported by the synergistic growth defect observed in *ΔnirB* and *Δhcp* double mutants, indicating their non-overlapping functions in mitigating nitrosative stress during nitrate respiration [33]. These findings collectively established NirBD and Hcp as central regulators of NO dynamics during respiratory nitrate utilization.

Beyond the canonical role of the NirBD enzyme, it may be involved in the process of N_2_O production in some microorganisms. For example, despite the strain *Rhodococcus* sp. S2 absence of typical denitrification genes, N_2_O production was observed during NO_3_^−^ reduction [34]. The amounts of N_2_O produced varied across microorganisms, ranging from 0.40 ± 0.06 μmol N_2_O (0.4 % of the added NO_3_^−^) for *Shewanella* sp. to 3.5 ± 0.3 μmol N_2_O (3.5 % of the added NO_3_^−^) for *Citrobacter* sp., both of which were reported to possess the *nirBD* gene [35]. N_2_O transiently reached 24 ± 14 μmol vial^−1^ (10 %–38 % of the added NO_3_^−^) in the type strain *Pseudomonas aeruginosa* PAO1-UW, which contains typical denitrification genes *nirS* and *nosZ* [36]*.* These findings suggested that the contribution of *nirS* to N_2_O emissions was 3–10 times greater than that of *nirBD*. Tong et al. [12] reported that kitchen waste oil (KFOG) as the carbon source can up-regulate the expression of *nar, nirB, nirD, norR* in the *Pseudomonas* CYCN–C, thereby achieving a total nitrogen (TN) removal efficiency of 73.5 %, which was higher than that of 60.9 % observed with sodium acetate. Specifically, the expression levels of *nirB* and *nirD* in the KFOG group were 30-fold and 49-fold higher than those in the sodium acetate group [12].

### 2.2 Functional analysis of NirBD in fungi

**Fig. 2:**
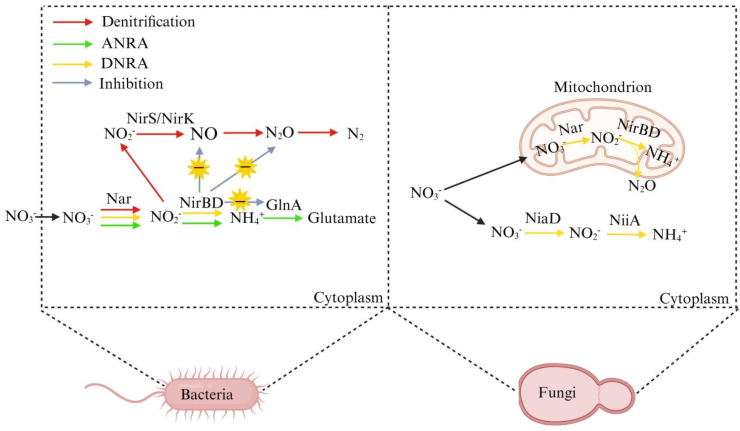
Comparison of the functions of NirBD in bacteria and fungi.

The DNRA process by fungi, also known as ammonia fermentation, is another form of fungal respiration**.** This process comprises three core biochemical processes: (1) the assimilation and step-wise reduction of nitrate to ammonium (2) the coupled oxidation of electron donors (e.g., ethanol), to acetate, and (3) substrate-level phosphorylation, which enables anaerobic growth. [37]. As shown in Fig. 2, the assimilatory nitrate and nitrite reductases (encoded by the *niaD* and *niiA* genes) are located in the fungal cytoplasm, which involved in the ammonia fermentation process. The assimilatory nitrate and nitrite reductases can utilize NADH or NADPH as electron donors to successively reduce nitrate to nitrite and then to ammonium [38]. Meanwhile, in the mitochondria, nitrite is produced from the reduction of nitrate by nitrate reductases such as NarA and/or NarB. Subsequently, nitrite is reduced to ammonium by nitrite reductase NirBD and/or NrfA [39]. In the ammonia fermentation of fungi, the reduction of nitrate is combined with the oxidation of ethanol or acetate through substrate-level phosphorylation [38]. The energy conservation mechanism uniquely integrates nitrate reduction with ethanol oxidation via a three-enzyme cascade: alcohol dehydrogenase (Ald), acetaldehyde dehydrogenase (AddA), and acetate kinase (Ack). Specifically, this pathway generates ATP through substrate-level phosphorylation while converting ethanol to acetic acid, with the liberated electrons driving nitrate ammonification [40]. During this process, a small amount of N_2_O was generated. Furthermore, this co-production was exemplified by *Aspergillus terreus*, isolated from the Arabian Sea, which simultaneously released N_2_O and NH_4_^+^ during the process of nitrate reduction under anaerobic conditions [41]. This physiology distinguished it from canonical bacterial DNRA, which was defined by the absence of both coupling to organic acid oxidation and dedicated N_2_O emission pathways.

### 2.3 Functional analysis of NirBD in actinomycetes

Actinobacteria, a ubiquitous soil-dwelling prokaryotic microorganisms, hold significant biotechnological importance due to their exceptional capacity to produce commercially valuable secondary metabolites including antibiotics, anticancer agents, and immunosuppressants [42]. Recent studies have revealed intriguing functional diversity in the nitrite reductase NirBD system across actinobacteria and bacterial taxa. In *Escherichia coli* and related enteric bacteria, *nirBD* expression is strictly anaerobic, whereas *Streptomyces coelicolor* uniquely encodes this operon under aerobic conditions [43]. Structural analyses demonstrated remarkable conservation between the *S.* c*oelicolor* and *E.* coli NirBD systems, exhibiting 52 % and 56 % amino acid sequence identity for their respective large (NirB) and small (NirD) subunits [44]. However, regulatory mechanisms diverge significantly across species. While *Mycobacterium tuberculosis* employs GlnR-mediated regulation of NirBD under nitrogen-limited conditions [45], *Streptococcus pneumoniae* exhibits a novel GlnR-independent nir expression pathway. This GlnR-independent nir expression system plays dual physiological roles by maintaining nitric oxide homeostasis through nitrite removal and facilitating nitrogen assimilation. Moreover, emerging evidence suggested GlnR-independent nir expression may also participate in interspecies signaling coordination via NO mediated metabolic synchronization [46]. Comparative studies highlight the superior performance of actinobacterial DNRA. *Streptomyces mediolani* EM-B2 exhibited a significant capacity for ammonium production. When nitrate was used as the nitrogen source, the production rate of ammonium reached 0.316 mg/L/h, which was more than threefold higher than the 0.095 mg/L/h observed in *Pseudomonas putida* Y-9 under identical carbon source conditions [47]. This result highlighted the superior performance of actinobacterial in the process of DNRA. The advantages in both temporal efficiency and the inherent catalytic effectiveness of actinobacterial NirBD systems, compared to their proteobacterial counterparts, endowed actinobacteria with distinct advantages, making them prime candidates for DNRA applications. Despite this potential, current understanding of the molecular mechanisms and comprehensive regulatory networks governing actinobacterial DNRA remains elusive.

## 3 Effects of NirBD-harboring microorganisms on the soil nitrogen storage and N_2_O emission

While public and research discourse often centers on the global carbon balance, the terrestrial nitrogen cycle has in fact been altered by human activities to a far greater extent. In the past century, the global warming potential of N_2_O has been 300 times that of CO_2_ [48]. The current environmental pressures from nitrogen pollution are primarily amplified by the two primary anthropogenic sources: excessive application of nitrogen-based fertilizers exceeding plant assimilation capacity, and uncontrolled discharge of nitrogen-rich industrial effluents [49]. The resultant nitrogen enrichment in aquatic systems can ultimately culminate in eutrophication, initiating a positive feedback loop that severely degrades water quality and ecosystem health [50]. The nitrogen cycle involves complex interconversions among various nitrogen species, including ammonium (NH_4_^+^), nitrate (NO_3_^−^), nitrite (NO_2_^−^), molecular nitrogen (N_2_), nitric oxide (NO), hydroxylamine (NH_2_OH), and hydrazine (N_2_H_4_). These transformations are mediated by diverse microorganisms in the environment [51]. The NirBD enzyme complex, serving as a critical metabolic nexus in both DNRA and denitrification process, is encoded by microorganisms ubiquitous in terrestrial and aquatic environments, thereby positioning these organisms as the central players in the global nitrogen cycle. There is an urgent need for methods to retain inorganic nitrogen in soils while removing it from water bodies. In soils, the NirBD enzyme complex can drive the conversion of nitrate/nitrite to ammonium, a process that conserves nitrogen within the ecosystem and mitigates nitrous oxide (N_2_O) emissions [49], which presents a viable strategy to mitigate the nitrogen loss.

### 3.1 Main NirBD-harboring microorganisms

Principal nitrogen storage microorganisms in soil comprise species from *Pseudomonas*, *Bacteroides*, *Methylobacteria*, *Aspergillus*, *Bacillus*, *Corynebacterium genera*, *Arthrobacter Dizzonia genus*, and *Microbacterium* [52]. Contrasting with terrestrial environments, aquatic systems demonstrate distinct nitrogen-cycling communities dominated by Cyanobacteria (particularly *Planktothrix*, *Anabaena*, and *Dolichospermum* genera), followed by Proteobacteria and Actinobacteria. Non-cyanobacterial genera with high relative abundances in the water included actinobacterial *Streptomyces, Planktophila*, gammaproteobacterial Pseudomonas and betaproteobacterial *Burkholderia* [53]. Facultatively anaerobic Listeria species are Gram-positive, non-sporulating rods ubiquitous in soil, aquatic systems, and food processing environments. The remarkable metabolic plasticity of NirBD-harboring microorganisms, a trait of profound environmental significance, is governed by oxygen-responsive genetic regulation that directly controls NirBD expression [54]. High-throughput sequencing analysis unraveled that Planctomycetota (3.65 %) had a high abundance in the anoxic zone of PIHSBBR, the nitrogen cycle function gene with the highest abundance was *nirBD* [55]*.* As major components of gasoline and petroleum, mono-aromatic hydrocarbons represent one of the most prevalent groups of contaminants in groundwater, sediments, soils, and industrial wastewaters associated with petroleum exploration and related industries [56] The genus *Aromatoleum* harbors the *nirBD*-type gene for nitrate reduction to ammonium, enabling the anaerobic degradation of mono-aromatic hydrocarbons [57]. The functional trait of NirBD confers a selective advantage in environments where hydrocarbon and nitrogenous pollutants co-occur, thereby positioning these microorganisms as the promising agents for targeted bioremediation. The widespread cultivability of *nirBD*-expressing microorganisms provides a practical advantage, enabling the rational design of targeted microbial consortia for simultaneous bioremediation of nitrogenous and organic pollutants. The key environmental microorganisms that harbor the NirBD enzyme are presented in Table 1.

**Table 1:** NirBD-harboring microorganisms and their distribution.

Species	Family	Habitat	Reference
*Pseudomonas putida*	Pseudomonadacea	Soil, waste water	[58]
*Staphylococcus argenteus*	Staphylococcaceae	Soil, rhizosphere	[59]
*Mycobacterium tuberculosis*	Mycobacteriaceae	Soil, water	[60]
*Bacillus cellulosilyticus*	Bacillaceae	Soil, rhizosphere	[61]
*Arthrobacter bambusae*	Arthrobacter	Soil	[62]
*Nocardia*	Nocardiaceae	Soil, beach sand	[63]
*Streptomyces griseus*	Streptomycetaceae	Soil, water sediment	[64]
*Burkholderia cepacia*	Burkholderiaceae	Water, soil	[65]
*Acinetobacter parvus*	Acinetobacter	Soil, water	[66]

### 3.2 Effects of NirBD-harboring microorganisms on soil nitrogen storage

**Fig. 3:**
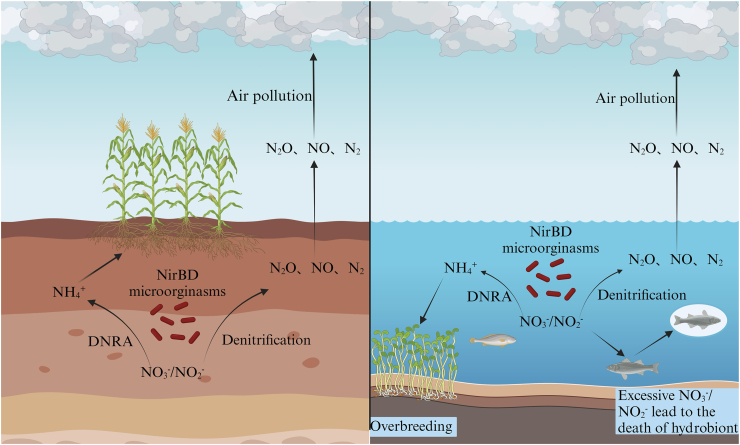
The effect of NirBD on soil nitrogen retention and N_2_O emissions.

Despite their predominance in the soil nitrogen (Fig. 3), the anionic nature of nitrate and nitrite however limits their direct and efficient uptake by plants. Consequently, the nitrogen use efficiency in conventional agriculture remains startlingly low, with crop assimilation typically capturing only 30–50 % of applied fertilizers, resulting in massive environmental nitrogen pollution [67]. This unassimilated nitrogen is primarily lost via leaching of soluble forms into aquatic systems or through microbial conversion into gaseous nitrogen oxides that escape to the atmosphere [68]. These nitrogen oxide gases pose significant environmental threats by acting as potent greenhouse gases and contributing to the depletion of the stratospheric ozone layer. Additionally, the influx of elevated nitrate and nitrite levels into water bodies triggers eutrophication, which in turn stimulates massive algal blooms. The subsequent decomposition of this algal biomass depletes dissolved oxygen, ultimately leading to widespread aquatic animal mortality [16].

Recently, the DNRA pathway has attracted significant research interest for its potential to enhance soil nitrogen availability. DNRA has three major advantages: preserving nitrogen, significantly reducing nitrate leaching and lowering N_2_O gas emissions. Through the NirBD enzyme, DNRA microorganisms can catalyze the reduction of nitrate/nitrite to ammonium in soils. Since the nitrogen is not incorporated into microbial biomass, it is retained in the environment as NH_4_^+^, a readily assimilable form for primary producers [13]. Although some DNRA microbes release small quantities of N_2_O during nitrate or nitrite reduction, the pathway remains an important mechanism for nitrogen retention in soil ecosystem [69]. Multiple NirBD-harboring microorganisms affect the nitrogen balance in oceans, estuaries, lakes, and land through DNRA [15]. The rate of DNRA varies depending on the type of ecosystem. Research has shown that the average DNRA rate in paddy soil is 1.30 mg N kg^−1^ day^−1^ ± 0.59, significantly higher than that in forests (0.24 mg N kg^−1^ day^−1^ ± 0.03), grasslands (0.52 mg N kg^−1^ day^−1^ ± 0.15), and the unfertilized farmland (0.18 mg N kg^−1^ day^−1^ ± 0.04). The highest DNRA rates were observed in humid subtropical regions (0.43 mg N kg^−1^ day^−1^), tropical humid zones (0.39 mg N kg^−1^ day^−1^), and coastal areas of ocean areas (0.39 mg N kg^−1^day^−1^), followed by the Mediterranean region (0.11 mg N kg^−1^day^−1^) [70]. These findings suggested that a warm and humid environment may be more suitable for the survival of DNRA microorganisms. There are also a large number of denitrifying microorganisms in the soil, which can reduce nitrate to nitrogen gas via denitrification, resulting in nitrogen loss [71]. Therefore, strategically enhancing DNRA activity while suppressing denitrification in surface soils emerges as a promising strategy to mitigate nitrogen loss and enhance crop nitrogen fertilizer efficiency. The metabolic function of NirBD-harboring microorganisms dynamically shifts between DNRA and denitrification, a process regulated by a suite of interconnected factors such as carbon-to-nitrogen (C/N) ratio, organic carbon availability, and ambient redox conditions [72]. The activity of DNRA could be promoted under conditions that characterized by low redox potential, high C/N ratios, available fermentable carbon, and significant concentrations of S^2−^ and Fe^2+^.

### 3.3 Effects of NirBD-harboring microorganisms on N_2_O emission

The enzymatic activity of NirBD in denitrifying microorganisms is a key regulator of N_2_O emission in aquatic ecosystems, especially under increasing anthropogenic pressures [73]. Rapid industrialization and continuous urbanization have increased wastewater discharge in aquatic ecosystems. More than 80 % of the wastewater generated globally is discharged without satisfactory treatment [74]. These untreated wastewater effluents are characterized by high concentrations of organic matter, nitrogen, phosphorus and heavy metals, posing substantial risks to aquatic organisms [75]. Projections indicated that the urban wastewater volumes will be increased by 24–50 % between 2030 and 2050, thereby exacerbating the nitrogen load. These inputs primarily take the forms of NO_3_^−^ and NO_2_^−^, which drive eutrophication cascades that progress from algal blooms to hypoxic and toxic byproduct formation [76]. While natural denitrification processes in estuarine sediments can remove nitrogen via N_2_ release, current anthropogenic nitrogen fluxes exceed the capacity of these biogeochemical pathways [77].

The environmental impact of NirBD stems from its catalytic function. By redirecting nitrite into the denitrification pathway, the downregulation expression of *nirBD* gene acts as a key control point that governs the flux of the potent greenhouse gas N_2_O. Multiple technologies are available for nitrate removal from water, including ion exchange, electrodialysis, reverse osmosis, adsorption, and electrocoagulation [78]. However, their practical application is often limited by high operational costs and challenges associated with disposing of concentrated waste streams such as brine [79]. In comparison, biological denitrification achieves high nitrate removal efficiency, up to 92.8 %, through sequential microbial reduction of nitrate to nitrogen gas (NO_3_^−^ → NO_2_^−^ → NO → N_2_O → N_2_) [80]. The application of mixed microbial consortia further enhanced process performance. For instance, the *Hanseniaspora uvarum* KPL108 demonstrated a nitrate removal rate of 9.37 mg/L/h with 92 % conversion to gaseous products [81]. Nevertheless, the presence of DNRA-competing microorganisms can restrict nitrate and nitrite availability for denitrifiers, while NirBD-mediated reactions directly influence N_2_O flux. Importantly, Yin et al. [82] demonstrated that *nirBD* gene expression regulated the critical metabolic branch point between N_2_O emission and its further reduction to N_2_, establishing this enzyme system as a vital control point for greenhouse gas mitigation [82].

The targeted regulation of NirBD activity represents a viable strategy for mitigating N_2_O emissions. This approach involves minimizing its contribution to DNRA while leveraging its function in denitrification to channel nitrogen intermediates towards N_2_, thereby enabling complete reduction and minimizing greenhouse gas release [83]. The optimization of NirBD activity directly enhanced the water quality by augmenting flocculation, restoring alkalinity, and lowering the operational costs of cBOD treatment [84]. A comprehensive understanding of microbial niche competition and enzymatic regulation is therefore crucial for designing integrated wastewater treatment systems that mitigate both eutrophication and the environmental impact of N_2_O emissions.

## 4 Expression mechanism of the *nirBD*

**Fig. 4:**
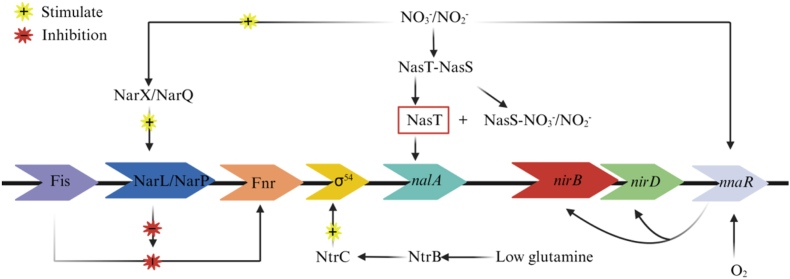
Expression model of *nirBD* operon.

As shown in Fig. 4, transcriptional regulation of the *nirBD* operon is mediated by multiple upstream regulatory elements. Expression from *nirB* and *nirD* requires both the Fnr protein and either NarL or NarP. Under hypoxia conditions, the global transcriptional regulator Fnr could be activated. Simultaneously, environmental nitrate or nitrite triggers phosphorylation of the response regulators NarL and NarP via the sensor kinases NarX and NarQ [85]. In addition to inhibiting the Fnr-mediated transcriptional activation, Fis may also bind additional proteins at downstream DNA sites within the regulatory pathway [86]. The major role of NarL and NarP is to counteract proteins that suppress Fis activity, thereby alleviating Fis-mediated inhibition of Fnr and permitting Fnr-dependent transcriptional activation. NasT, as an essential antiterminator protein, is required for the expression of the *nirBD* operon, which encodes nitrite reductase. In the absence of NasT, the leader RNA (NalA) of the *nirBD* operon forms a stable hairpin that blocks transcription elongation [87]. NasT, as a member of the ANTAR antiterminator family, regulates the transcription through RNA binding. NasS belongs to the small-molecule-binding protein superfamily, which often associated with ABC-type transport systems. When nitrate/nitrite are absent, NasS binds tightly to NasT, thereby inhibiting its antitermination activity. In the presence of nitrate or nitrite, the NasS-NasT complex undergoes a conformational change that releases NasT, which then activates target gene transcription by binding to cognate RNA transcripts [88,89]. NasT binds NalA with high affinity, activating its antitermination function and permitting normal transcription of the *nirBD* operon. The transcription of *nalA* is initiated by the σ^54^-dependent promoter and mediated by phosphorylation of the response regulator NtrC. The formation of transcriptionally competent open complexes at the σ^54^-dependent promoter requires phosphorylation of NtrC. Under nitrogen-limiting conditions, the sensor kinase NtrB autophosphorylates and transfers the phosphate group to NtrC. The phosphorylation state of NtrC is regulated by nitrogen availability: it is unphosphorylated with preferred sources like ammonium or glutamine, but phosphorylated when glutamate, nitrate, or nitrite are provided [90]. The orphan response regulator *nnaR* regulated nitrate/nitrite assimilation. Originally identified as a GlnR target in *Streptomyces coelicolor*, NnaR acted synergistically with GlnR to co-activate the expression of *nirB*, *narK* and *nasA* [91]. While the NnaR-associated operon was suppressed under hypoxia, it was strongly induced by inorganic nitrogen sources, particularly nitrate and nitrite [92]. NnaR_Mab_ activated the transcription of genes involved in nitrate reduction to nitrite (NasN), nitrite reduction to ammonium (NirBD), nitrate/nitrite transport (NarK3), and the synthesis of the nitrite heme cofactor reductase [93]. Under normal conditions, these components act coordinately to ensure efficient transcription of the *nirBD* operon.

## 5 The influence of different factors on *nirBD* expression

### 5.1 c-di-GMP and NasT modulate the suppression of *nirBD* expression

The acquisition of intracellular nitrate prior to its reduction is mediated by the Nas enzyme system and its associated ABC-type ATP-dependent transporters, facilitated by periplasmic binding proteins that ensure substrate specificity [94]. This transport system is integral to nitrate uptake and central to the regulatory network governing *nirBD* expression. A key regulatory mechanism involves the NasT antiterminator, which binds to the leader RNA (NalA) of the *nirBD* operon. In the absence of NasT, NalA forms a transcription-blocking hairpin structure via base-pairing between its 5′- and 3′- regions. Binding of NasT induces structural remodeling of NalA, specifically disrupting the terminator-associated T-loop and permitting transcriptional elongation [95]. Cyclic diguanylate (c-di-GMP), a ubiquitous bacterial second messenger, downregulates *nirBD* expression by interfering with the NasT-NalA interaction. When c-di-GMP is present, it binds to NasT, forming a NasT-c-di-GMP complex. The obtained NasT-c-di-GMP complex exhibits reduced affinity for NalA, resulting in transcriptional termination [87]. Therefore, c-di-GMP acts as a negative regulator of nitrite reductase activity in a NasT-dependent manner.

### 5.2 HrcA inhibits *nirBD* expression by suppressing the activity of chaperone protein

The functional assembly of the nitrite reductase complex NirBD depends on gro*EL*/*groES* chaperone-mediated folding, a process controlled through proteasome-regulated degradation of the transcriptional repressor HrcA. HrcA, a conserved bacterial repressor protein, regulates chaperones of the Hsp60 family by directly binding to the promoters of three chaperonin-encoding operons (*groES*, *groEL1*, *groEL2*), thereby suppressing their expression [96]. Proteasomes mediated degradation of HrcA to relieves this repression, enabling *groEL*/*groES* expression and supporting NirBD activity through proper cofactor incorporation [97]. In *Mycobacterium tuberculosis*, certain proteasome substrates are covalently modified with prokaryotic ubiquitin-like protein (Pup) by the ligase PafA (proteasome cofactor A) [98]. Pup-tagged proteins are recognized by the mycobacterial proteasome ATPase (Mpa), which uses ATP hydrolysis to unfold substrates and translocate them into the 20S core particle (20S CP) for degradation [99]. Therefore, in *Mycobacterium tuberculosis*, HrcA represses the chaperonin genes *groES*, *groEL1*, and *groEL2*, indirectly limiting the expression of *nirBD*. Degradation of HrcA by the Mpa/20S proteasome restores chaperonin production, thereby promoting the folding and assembly of functional NirBD.

### 5.3 Other factors

The expression of NirBD in microorganisms is influenced by multiple environmental and nutritional factors. Key parameters include pH, temperature, carbon source, rotational speed, and carbon-nitrogen ratio, each affecting enzymatic activity and gene regulation. Table 2 summarizes the major factors known to modulate *nirBD* expression and their optimal ranges for microbial growth and activity.

**Table 2:** Factors affecting NirBD in microorganisms and the optimal values.

Species	T/°C	C sources	C/N ratio	pH	Shaking speeds	Reference
*Pseudomonas putida* Y-9	–	Glucose	9	7	150 r/min	[100]
*Pseudomonas mosselii* 9-	20	Sodium succinate	18	6,7,9	–	[101]
*Pseudomonas* sp. XS-18	25	–	13	10,11	–	[102]
*Alcaligenes faecalis* strain WT14	20	–	14	7	104 r/min	[103]

— not mentioned.

## 6 Conclusions and future prospects

The transformation of NO_3_^−/^NO_2_^−^ in soil ecosystems is critically mediated by the microorganisms harboring the NirBD enzyme, which governs the balance between nitrogen retention and greenhouse gas emissions. Our comprehensive analysis indicated that NirBD could act as a key biochemical switch in nitrogen cycling pathways: converting NO_3_^−/^NO_2_^−^ to NH_4_^+^ via fermentative DNRA, while also modulating N_2_O emission fluxes. The *nirBD* gene expression is tightly regulated by metabolic demands, which in diverse bacterial and fungal species typically requires elevated nitrate levels and occurs mainly under anaerobic or microaerophilic conditions. This dual metabolic role has been well characterized in *Pseudomonas putida*. Mechanistic studies further revealed that *nirBD* expression is controlled by sophisticated regulatory networks responsive to redox state and substrate availability, underscoring its role as both a metabolic effector and an environmental sensor in nitrogen transformations. Ecologically, NirBD-containing microorganisms contribute not only to soil nitrogen conservation but also to aquatic bioremediation, where their activity governs the interconversion rates of the key nitrogen forms, including NO_3_^−^, NO_2_^−^, NH_4_^+^ and N_2_O. In summary, NirBD represents a promising yet underexplored enzymatic target. To fully realize its potential, future research should focus on the specific areas outlined below.

(1) The function of NirBD enzyme-encoding genes in microbial nitrogen metabolism should be further investigated with gene knockout techniques like CRISPR/Cas9. This approach will elucidate the molecular mechanisms and pathways underlying nitrogen conversion.
(2) Most of the functions of NirBD discovered so far were mainly elucidated from bacteria. In the future, more research on the functions of NirBD should be conducted in fungi and actinomycetes. This will deepen our understanding of the nitrogen cycling mechanism in microorganisms, which is beneficial for soil nitrogen retention and lowering the N_2_O emissions.
(3) NirBD activity is influenced by multiple environmental factors, including temperature, pH, redox potential, carbon to nitrogen ratio, carbon source, and the presence of certain ions (Fe^2+^, S^2−^) in the environment. Targeted optimization of these parameters in soils and aquatic systems may offer a practical strategy for enhancing NirBD-mediated nitrogen retention.
(4) Future investigations should aim to elucidate the evolutionary conservation of NirBD enzyme, evaluate its substrate affinity under dynamic environmental conditions, and explore engineering strategies that decouple ammonium production from N_2_O emissions. Such advances hold significant potential for precise applications in sustainable agriculture and wastewater treatment, concurrently lowering associated climate effects.

## CRediT authorship contribution statement

**Yidan Peng:** Writing – original draft, Methodology, Investigation, Conceptualization. **Tengxia He:** Writing – review & editing, Resources, Project administration, Funding acquisition, Conceptualization. **Qimin Zhou:** Writing – review & editing. **Mengyuan Yin:** Writing – review & editing. **Chengtao Jin:** Writing – review & editing.

## Funding

This work was supported by 10.13039/501100001809National Natural Science Foundation of China (No. 42167019) and Scientific Research Innovation Team Project of 10.13039/501100003459Guizhou University [No. Guidakechuangtuan (2024) 06].

## Declaration of competing interest

The authors declare that they have no known competing financial interests or personal relationships that could have appeared to influence the work reported in this paper.
